# Synergism of rMV-Hu191 with cisplatin to treat gastric cancer by acid sphingomyelinase-mediated apoptosis requiring integrity of lipid raft microdomains

**DOI:** 10.1007/s10120-021-01210-8

**Published:** 2021-07-12

**Authors:** Yao Lv, Chu-di Zhang, Yi-long Wang, Dong-ming Zhou, Meng-ying Zhu, Xiao-qiang Hao, Jin-hu Wang, Wei-zhong Gu, Hong-qiang Shen, Jin-gan Lou, Ben-qing Wu, Pei-chun Chen, Zheng-yan Zhao

**Affiliations:** 1 Gastroenterology Department, Children’s Hospital, Zhejiang University School of Medicine, Hangzhou, 310051 Zhejiang China; 2 Children’s Hospital, Zhejiang University School of Medicine, Hangzhou, 31005 Zhejiang China; 3 University of Chinese Academy of Sciences, Shenzhen Hospital, Shenzhen, 518000 China

**Keywords:** Gastric cancer, Recombinant Chinese Hu191 measles virus, Cisplatin, ASMase, Lipid rafts

## Abstract

**Background:**

DDP-based chemotherapy is one of the first-line treatment in GC. However, the therapeutic efficacy of DDP is limited due to side effects. Therefore, it is of great significance to develop novel adjuvants to synergize with DDP. We had demonstrated previously that rMV-Hu191 had antitumor activity in GC. Here we examined the synergism of rMV-Hu191 with DDP in vitro and in vivo.

**Methods:**

Cellular proliferation, the synergistic effect and cell apoptosis were evaluated by CCK-8 assay, ZIP analysis and flow cytometry, respectively. The protein levels and location of ASMase were monitored by western blot and immunofluorescence assay. shRNA and imipramine were used to regulate the expression and activity of ASMase. MβCD was administrated to disrupt lipid rafts. Mice bearing GC xenografts were used to confirm the synergism in vivo.

**Results:**

From our data, combinational therapy demonstrated synergistic cytotoxicity both in resistant GC cell lines from a Chinese patient and drug-nonresistant GC cell lines, and increased cell apoptosis, instead of viral replication. Integrity of lipid rafts and ASMase were required for rMV-Hu191- and combination-induced apoptosis. The ASMase was delivered to the lipid raft microdomains at the initial stage of rMV-Hu191 treatment. In vivo GC mice xenografts confirmed the synergism of combinational treatment, together with increased apoptosis and trivial side-effects.

**Conclusions:**

This is the first study to demonstrate that rMV-Hu191 combined with DDP could be used as a potential therapeutic strategy in GC treatment and the ASMase and the integrity of lipid rafts are required for the synergistic effects.

**Supplementary Information:**

The online version contains supplementary material available at 10.1007/s10120-021-01210-8.

## Introduction

Gastric cancer (GC) is the fifth most common malignancy worldwide, and its overall mortality rate is the third highest in all types of cancers [1, 2]. Currently, the main therapeutic methods used to treat GC include surgery, radiotherapy, chemotherapy and neoadjuvant therapy [3]. First-line treatment of advanced gastric cancer is based on cisplatin (DDP) and 5-fluorouracil [4]. However, the therapeutic efficacy of DDP treatment to GC patients is limited due to the toxicity induced by the relatively high dose applied in conventional chemotherapy, as well as the frequent development of drug resistance [5, 6]. Therefore, novel adjuvants are always desired with hope to increase the potency of the drugs, to reduce the side effects, and to counteract chemoresistance.

Combining therapeutic modalities have an ability to produce additive or synergistic effect exceeding either approach alone. Combination of oncolytic virotherapy, chemotherapy, or immunotherapy has been investigated in recent pre-clinical and clinical studies to maximize the efficacy [7–10]. Measles virus (MV), one of the oncolytic viruses, has been investigated as a new treatment modality against a number of tumor entities including hepatocellular carcinoma, breast cancer, pancreatic cancer, and colon cancer [11–14]. Several oncolytic MV strains had been undergone clinical trials as antitumor agents [15, 16]. Phase I/II clinical trials had shown promise but treatment efficacy needs to be enhanced, otherwise might endanger its broad clinical success [17]. Furthermore, previous studies showed that combinational treatment with oncolytic measles vaccine viruses enhanced killing of therapy-induced senescent tumor cells including hepatoma, pancreatic cancer, and mammary gland carcinoma with chemotherapeutics including gemcitabine, doxorubicin, and paclitaxel [18]. Oncolytic MV in combination with chemotherapeutics shows promising prospects, since such regimens could efficiently eliminate both primary tumors and metastases.

The safety of the measles vaccine has been proved worldwide in controlling measles [19], and the live-attenuated Chinese Hu191 measles virus strain has the same safety record for 50 years [20]. Recently in our laboratory, rMV-Hu191, a recombinant Hu191 strain of measles virus has been successfully established [21]. It has been found in previous studies that rMV-Hu191 alone could be used as a potentially effective therapeutic agent in gastric cancer and colorectal cancer treatment [22, 23]. Whether the rMV-Hu191 could synergize with DDP in GC is not reported to the date. This study evaluated the antitumor activity of rMV-Hu191 combined with DDP against two drugs nonresistant human GC cell lines and one multi-drug-resistant signet ring cell line from a Chinese male signet ring cell gastric cancer patient and mice bearing xenografts. Furthermore, the underlying mechanisms of the combinational therapy were discussed.

Apoptosis, one type of cell death models, is mainly regulated by intrinsic, extrinsic apoptotic pathways and pro-apoptotic lipid signaling pathways [24, 25]. We have previously shown that rMV-Hu191 alone could induce apoptotic cell death requiring integrity of lipid raft microdomains in human gastric cancer [22]. The present study further explores the role of lipid rafts in rMV-Hu191-induced apoptosis. Lipid rafts as major platforms for signal regulation in cancer are high content of cholesterol and sphingomyelin (SM) membrane microdomains [26]. Acid sphingomyelinase (ASMase) is a lysosomal phosphodiesterase that catalyzes the hydrolysis of SM to produce phosphocholine and ceramide. Ceramide is a pro-apoptotic protein regulating a key initiative step in apoptosis [27]. The binding of microbial pathogens and membrane damage could lead to the activation of ASMase and translocation of ASMase from lysosome to the lipid raft microdomains, and the subsequent generation of ceramide promotes the initiation of the apoptosis [28]. Thus, we hypothesized that ASMase and lipid rafts were required for rMV-Hu191 inducing apoptotic cell death in GC cells.

Despite that DDP binds to nucleic acids, thiol-containing peptides, microfilaments, and membrane phospholipids, DNA is generally considered as the major biological target of DDP [29]. The main mechanism of DDP is through crosslinking DNA and the subsequent prevention of DNA replication and transcription, which ultimately leads to cell death through interconnections between apoptosis and necrosis pathways [30]. In literature reports, DDP could induce caspase-dependent apoptosis in GC [31, 32]. Therefore, we hypothesized that rMV-Hu191 could synergize with DDP to induce tumor cell apoptotic death.

The novel chemovirotherapy strategy being investigated here could be of clinical benefit by effectively reducing tumor burden and decreasing chemo-resistance together with toxicity. Furthermore, we uncovered a novel mechanism of virus-induced sensitization to DDP therapy.

## Materials and methods

### Cell culture and reagents

The BGC-823 and SGC-7901 cells were purchased from the Cell Bank of the Chinese Academy of Sciences, Shanghai, China, and the multi-drug-resistant signet ring cell GCSR1 [33] was a general gift of professor Li-song Teng. Cell morphology of GCSR1 in culture maintained in vitro for over 90 passages, is similar to the cells from the patient. The BGC-823 and GCSR1 cells were cultured in RPMI 1640 medium (Life Technologies, USA) supplemented with 10% FBS, and the SGC-7901 cells were supplemented with 20% FBS. African green monkey kidney Vero cells were purchased from the American Type Culture Collection (ATCC, USA), and cultured in DMEM (Life Technologies, USA) supplemented with 10% FBS. All cells were cultured at 37 °C with 5% CO_2_ and saturated moisture. Reagents: cisplatin (Sigma, P4394), Z-VAD-FMK (ApexBio, A1902), Methyl-β-cyclodextrin (Sigma, 332615), imipramine (ApexBio, C4117).

### Cell viability assays

BGC-823, SGC-7901 and GCSR1 cells were plated at 4 × 10^3^ or 3 × 10^3^ cells per well in 96-well plates. After 24 h, cells were treated with rMV-Hu191 at different multiplicity of infection (MOI), and cisplatin (DDP) was then added 24 h post infection. Cell viability was calculated based on standard curves by CCK8 assay (Dojindo Molecular Technologies, USA) at every 24-h interval.

### Zero interaction potency (ZIP) analysis

We determined the complete combination matrix for antagonism or synergy using the ZIP model. This new method is based on the hypothesis that two non-interacting drugs will simply affect their dose–response curves, but not the half maximal inhibitory concentration (IC50) or the shape of the curve. Therefore, any deviation in these values detected when combining with another drug can indicate antagonism or synergism [34, 35]. Data from individual dose response experiments were used to calculate the ZIP scores. For this analysis we used the R package “SynergyFinder 1.0.” (www.synergyfinder.fimm.fi).

### Virus construction, titration and replication assays

rMV-Hu191 was constructed as previously described [21]. Virus titers were calculated on Vero cells by the TCID 50 method [36]. For virus replication assays, cells were plated at 2 × 10^5^ per cell in six-well plates. After 24 h, cells were infected with rMV-Hu191 at MOI = 0.1 or MOI = 1 in Opti-MEM medium (Life Technologies, USA) for 2 h. Then the virus containing medium was replaced with fresh media. DDP was added at indicated dose 24 h post infection. Cells were harvested with supernatant at indicated time intervals. Then virus titers were measured by TCID 50 assay on Vero cells after two freeze–thaw cycles and centrifugation.

### Disruption and isolation of cholesterol-rich ‘lipid rafts’

Methyl-β-cyclodextrin (MβCD) was known as a compound to extract cholesterol from biomembranes. Drug stocks (50 mg/ml) were prepared in PBS and stored at 4 °C until use. To disrupt lipid rafts, cells were incubated with 7.5 mM MβCD 2 h prior to rMV-Hu191 infection and then 2 mM MβCD for 48 h after combinational treatment.

The lipid raft fractions were separated as previously described [22]. Briefly, GC cells after desired treatments were incubated with ice-cold solubilization buffer. After incubation, the sample was homogenized with needle and centrifuged at 500*g* for 5 min at 4 °C (Allegra 6R, Beckman Coulter, USA). The supernatant was mixed with TritonX-100 (Solarbio, China) at the final concentration of 0.5% (w/v, in solubilization buffer). The membrane fractions mixed with sucrose were centrifuged at 200,000*g* at 4 °C for 14 h (Optima ultracentrifuge, Beckman Coulter, USA). Then ten serial aliquots were collected from the top and labelled as 1–10. Finally, all fractions were detected by Western blotting as described below.

### Functional inhibition of ASMase

Imipramine as a specific ASMase inhibitor could block the function of ASMase. A 300 mM stock of imipramine was prepared in DMSO and stored at −20 °C. For ASMase functional inhibition assays, imipramine was incubated at 25 μM in BGC-823 cells and 50 μM in SGC-7901 cells for 72 h after rMV-Hu191 infection.

### Construction of ASMase-knockdown SGC-7901 cell clones

We used shRNA targeting the ASMase gene (SMPD1) to reduce ASMase expression and shCtrl as a negative control. shASMase and shCtrl plasmids obtained from Genechem (Shanghai, China) were packaged as lentiviral constructs using lipofectamine 2000 (Invitrogen, 11668027) and then transfected to the SGC-7901 cells. Stable transfectants were screened with puromycin (1:12,000) to obtain single cell clone for amplification. Protein levels of ASMase were measured by Western blotting.

### Flow cytometry analysis

A density of 1 × 10^6^ GC cells at indicated treatments were harvested, washed twice in PBS and stained with Annexin V FITC Apoptosis Detection Kit (BD Biosciences, 556547) following the manufacturer's instructions. Triplicates for each group were detected by a flow cytometer (Navios, Beckman Coulter, USA) and analyzed with Flowjo 10.0 software (Tree Star Inc, USA).

### Western blotting

Cells after desired treatment were harvested at indicated times, washed once with PBS, and then were lysed in RIPA buffer (Beyotime, P0013B) with phosphatase and protease inhibitors. 30 µg of proteins per well were separated by SDS-PAGE gel and electroblotted onto Immuno-blot PVDF membrane (Bio-Rad Laboratories, 1620177). The membranes blocking with 5% nonfat milk at room temperature for 1 h, were probed with primary antibodies at 4 °C overnight, followed by secondary antibodies at room temperature for 1 h. Then the membranes were visualized using an EZ-ECL detection kit (Biological Industries, 20-500-120). For Western blot analysis: anti-β-actin (CST, 4970S), anti-caspase-3 (CST, 9665S), anti-PARP (CST, 9542S), anti-Acid sphingomyelinase (ASMase, abcam, ab74281), anti-flotillin 1 (abcam, ab133497), secondary HRP-linked anti-rabbit IgG (CST, 7074S), secondary HRP-linked anti-mouse IgG (CST, 7076S).

### Immunofluorescence

BGC-823 and SGC-7901 cell lines were cultured in 24-well plates at 4 × 10^5^ cells per well. The cells after indicated treatment were gently washed with PBS, fixed in 4% paraformaldehyde (PFA) for 30 min, permeabilized by 0.1% TritonX-100, and blocked by 2% BSA. Cells were incubated with mouse anti-measles phosphoprotein (MV-P, abcam, ab43820), followed by Alexa Fluor^®^ 594 donkey anti-mouse IgG (H + L) (Invitrogen, A-21203) and DAPI (Beyotime, C1005) in the dark, images were captured using Zeiss cLSM780 microscope. For co-localization analysis, cells were stained with Alexa 488-conjugated CTB (Invitrogen, C-34775) for 2 h at 37 °C incubator before fixation by 4% PFA. Then cells were incubated with anti-Acid sphingomyelinase (ASMase, abcam, ab83354) at 4 °C overnight, followed by Alexa Fluor^®^⁠ 594 goat anti-rabbit IgG (H + L) (Invitrogen, A-11012) and DAPI in the dark. Images were captured using Lecia TCS SP8 confocal laser scanning microscope.

### In vivo antitumor studies

All institutional and national guidelines for the care and use of laboratory animals were followed. The animal experiments were handled according to the animal ethical committee of Zhejiang Chinese Medical University. 1 × 10^6^ BGC-823 cells suspended in 100 μl PBS were subcutaneously injected into the right flank of athymic nude mice (3–5 weeks, male) obtained and housed in the animal center of Zhejiang Chinese Medical University.

On day 5 post implantation, mice were randomly divided into four groups (*n* = 10) when tumor volume reached approximately 30–50 mm^3^ (*V* = length × width^2^/2).

From this day, mice were injected intratumorally with 1.4 × 10^7^ (TCID 50) of rMV-Hu191 suspended in 100 μl Opti-MEM six times on day 5, 6, 7, 9, 11 and 13 post implantation. Mice that received DDP treatment were intraperitoneally injected DDP at 10 mg/kg on days 7, 14, and 21. Control groups were scheduled to receive sham injections of Opti-MEM or PBS instead of injections of rMV-Hu191 or DDP. For animal survival, tumor volumes and weight were recorded every third day until day 11 post tumor implantation, and daily after day 11, mice were euthanized when tumor volumes exceeded 1500 mm^3^ or body weight loss over 20%.

### Evaluation of side-effects in vivo

On day 13 post implantation, three mice from each group were sacrificed to harvest tumor tissues for analysis of apoptosis and angular vein blood for analysis of liver and kidney functions. Angular vein blood was collected to perform liver and kidney functions on an automated blood chemical pipeline analyzer (Beckman Coulter, USA).

### Immunohistochemistry (IHC)

Tumor tissues were fixed in 4% formalin, embedded in paraffin, cut into 4 μm slices, and stained with hematoxylin and eosin (H&E) or indicated antibodies. Apoptotic cells in tumor sections were analyzed by an in situ Apoptosis Detection Kit (TUNEL, Takara, MK500). Color changes of the slides were photographed using light microscope with companion software (Leica, Germany).

### Statistical analysis

All values were performed at least three independent experiments. Data were reported as mean ± SE. Statistical significance between data sets was analyzed by Student’s *t* test and one-way ANOVA using GraphPad Prism 9.0. Survival analysis was calculated by the Kaplan–Meier method and compared by the log-rank test. *P* value < 0.05 was considered statistically significant.

## Results

### rMV-Hu191 synergistically enhanced the cytotoxicity of DDP in human GC cells

**Fig. 1:**
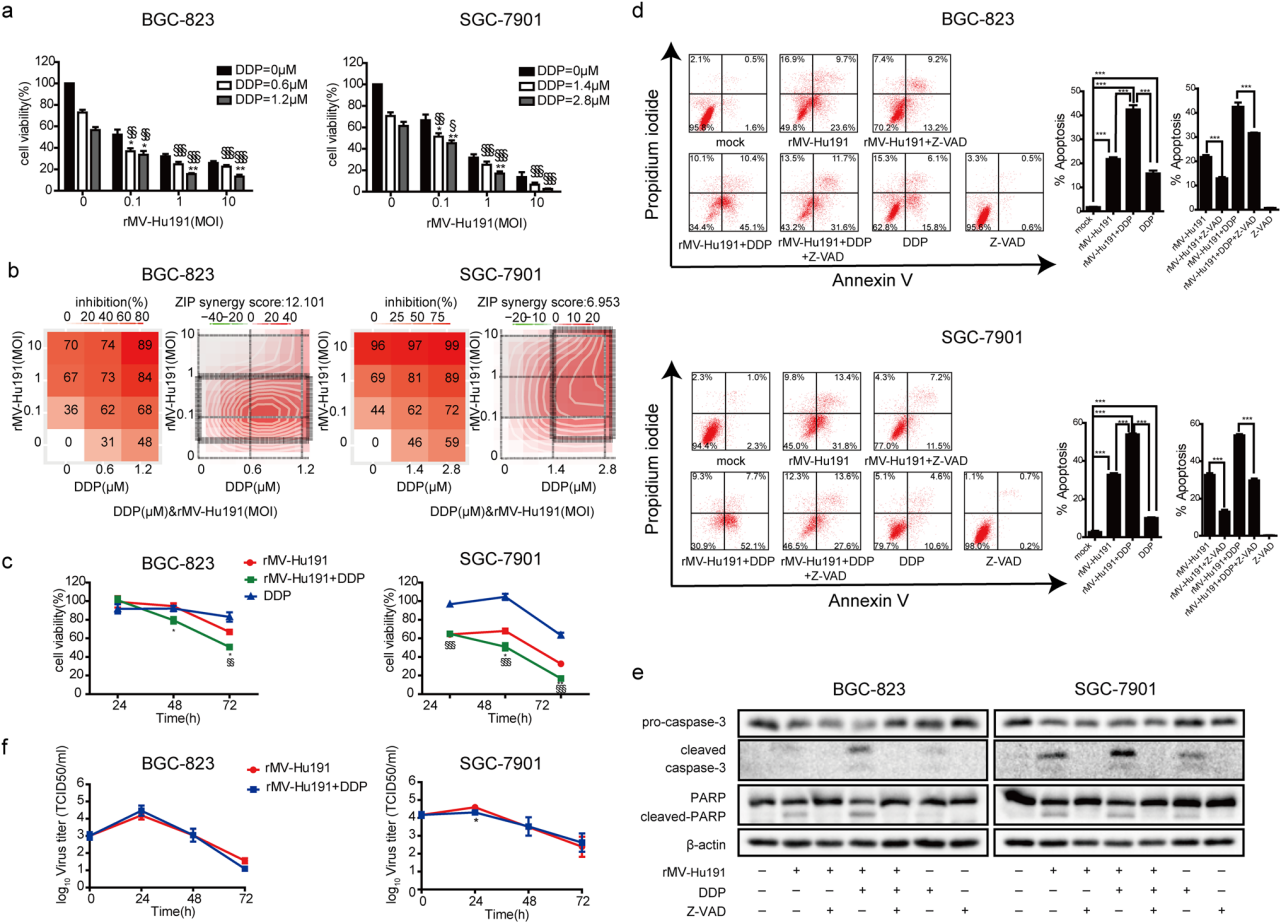
rMV-Hu191 and DDP synergistically enhanced the cytotoxicity and caspase-dependent apoptosis in human GC cells. **a** Viability of BGC-823 and SGC-7901 cells after combinational treatment with rMV-Hu191 at different MOIs and DDP at indicated doses for 72 h. Data presented as mean ± SE. **b** Inhibition and ZIP synergy score of rMV-Hu191 and DDP in BGC-823 and SGC-7901 cells. Circled area indicated the strongest synergistic region. **c** Viability of GC cells treated with rMV-Hu191 and DDP at different time points. MOI was 0.1 and 1 and the dose of DDP was 0.6 and 2.8 μM in BGC-823 and SGC-7901 cells, respectively. * indicated comparison between the combinational and rMV-Hu191 treated groups, *P < 0.05, **P < 0.01, ***P < 0.001 (one-way ANOVA); ^§^ indicated comparison between the combinational and DDP treated groups, ^§^*P* < 0.05, ^§§^*P* < 0.01, ^§§§^*P* < 0.001 (one-way ANOVA). **d** Ratio of apoptotic cells by flow cytometry analysis and **e** expression of apoptosis marker proteins pro-caspase 3, cleaved-caspase-3, PARP, and cleaved-PARP in BGC-823 and SGC-7901 cells treated at the pre-determined condition that permitted synergism, in the presence or absence of Z-VAD (100 μM) for 72 h. ***P < 0.001 (one-way ANOVA). **f** Virus particles were quantified by TCID 50 in GC cells treated with rMV-Hu191 alone or combination of rMV-Hu191 and DDP. *P < 0.05 (student’s *t* test)

As shown in Fig. 1a and Fig. s1, compared to treatment with rMV-Hu191 or DDP alone, their combination significantly augmented the cytotoxicity in GC cells. ZIP analysis confirmed that the interaction between rMV-Hu191 and DDP was almost universally synergistic over all the dose–response matrix in GC cells (Fig. 1b). For BGC-823 cells, the strongest synergistic effect was found within the region of dose combinations (MOI = 0.1 and DDP = 0.6 μM) with average ZIP synergy scores 12.101. As for SGC-7901, the strongest synergistic effect was found within the region of dose combinations (MOI = 1 and DDP = 2.8 μM) with average ZIP synergy scores 6.953. The time-dependent synergistic effect of rMV-Hu191 and DDP was initiatively detected at 48 h after combinational treatment with optimum doses according to the ZIP synergy scores (Fig. 1c).

### rMV-Hu191 and DDP cooperated to trigger caspase-dependent apoptosis without virus progeny alteration in GC cells

**Fig. 2:**
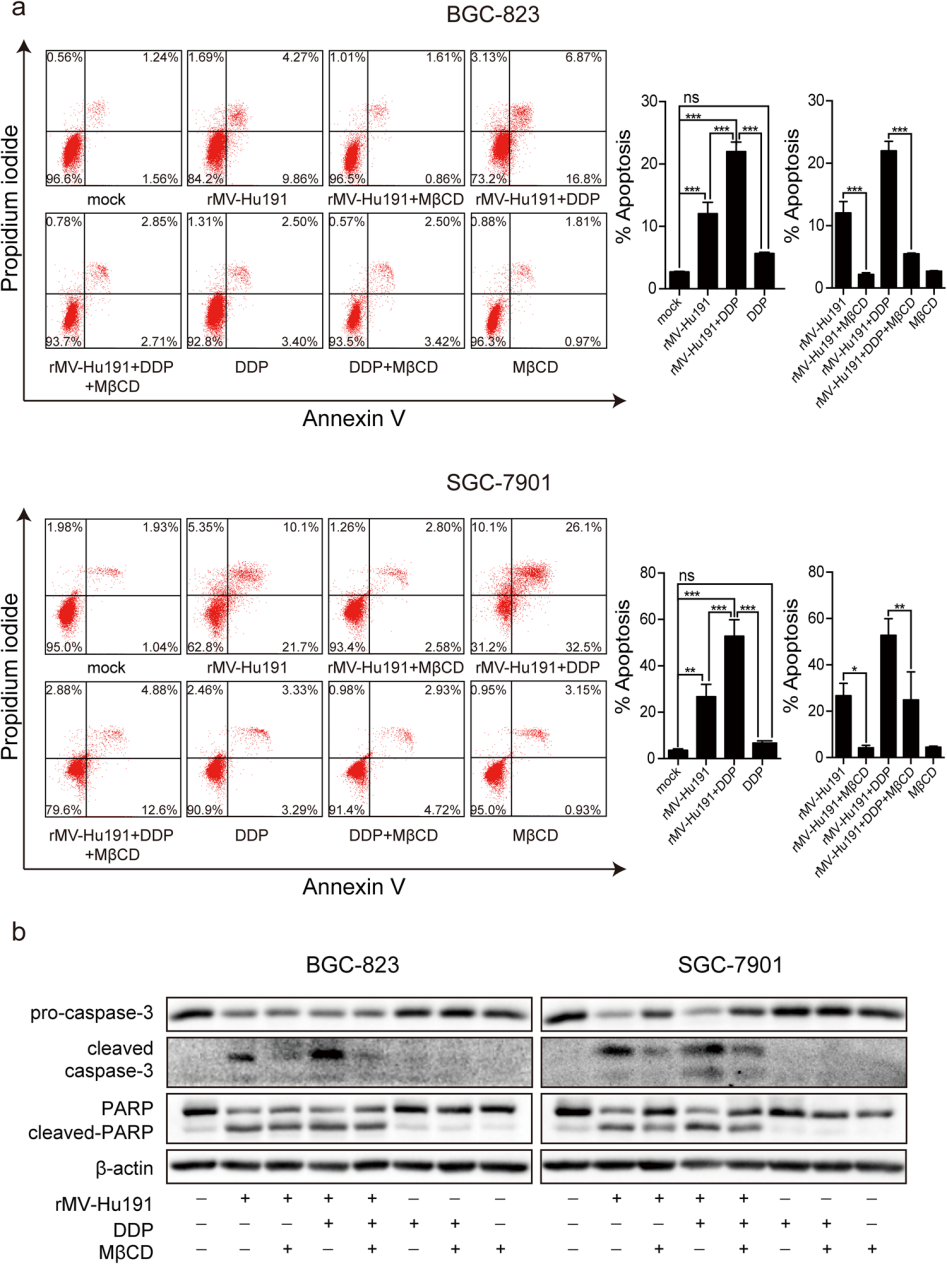
rMV-Hu191- and combinational-treatment induced apoptosis required lipid rafts integrity. **a** Ratio of cell apoptosis after rMV-Hu191 and DDP treatment for 48 h in BGC-823 (MOI = 0.1, DDP = 0.6 μM) and SGC-7901 (MOI = 0.1, DDP = 2.8 μM) cells, with or without MβCD treatment. **b** Expression of apoptotic proteins after the same treatment as above. *P < 0.05, **P < 0.01, ***P < 0.001

As shown in Fig. 1d, the current experiment exhibited that rMV-Hu191 in combination with DDP demonstrated a synergistic effect of caspase-dependent apoptosis which could be partially inhibited by Z-VAD (a pan-caspase inhibitor) in GC cells. The consistent results were confirmed by detecting the activation of apoptosis marker proteins caspase-3 and PARP (Fig. 1e). To explore whether the existence of DDP affected virus replication, we detected the virus growth curves. For both BGC-823 and SGC-7901 cells, there was almost no alternation in virus growth kinetics treated with rMV-Hu191 alone or combination with DDP (Fig. 1f, Fig. 2).

### Lipid rafts integrity was required for rMV-Hu191- and combination-induced apoptosis

Lipid rafts integrity was disrupted by MβCD treatment dissolving cholesterol from cell membranes which was confirmed by the alteration of the position of flotillin 1, a marker protein of lipid rafts (Fig. s3) [37]. As shown in Fig. s4, the cells density of rMV-Hu191 and combination treatment was prominently reversed by MβCD. And the proportion of apoptosis in rMV-Hu191 group and combination group was significantly reversed after lipid rafts disruption using MβCD (Fig. 2a). To further investigate lipid rafts requirement in the rMV-Hu191 and combination induced apoptosis, we detected the activation of caspase 3 and PARP. The cleavage of apoptosis proteins was remarkably decreased in the existence of MβCD (Fig. 2b). These results suggested the vital role of lipid rafts in the synergistic effect of rMV-Hu191 and combination.

### ASMase participated in the rMV-Hu191 and combinational treatment induced apoptosis

**Fig. 3:**
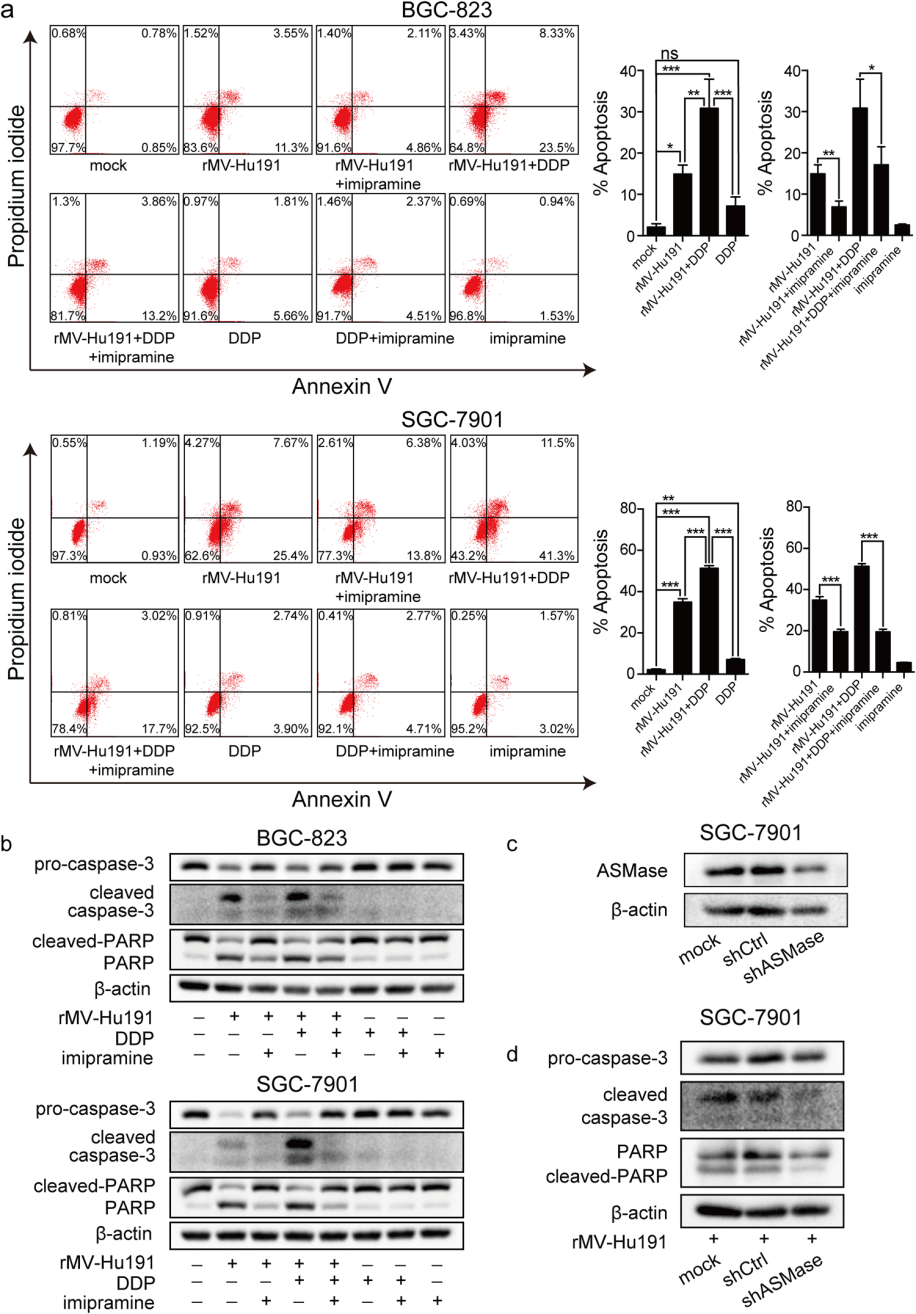
ASMase participated in rMV-Hu191- and combination-induced apoptosis. **a** Ratio of cell apoptosis after rMV-Hu191 and DDP treatment for 48 h in BGC-823 (MOI = 0.1, DDP = 0.6 μM) and SGC-7901 (MOI = 0.1, DDP = 2.8 μM) cells, with or without imipramine treatment. **b** Expression of apoptotic proteins after the same treatment as above. **c** Protein levels of ASMase in wild-type SGC-7901 cells and SGC-7901 cells stably transfected with shASMase or shCtrl. **d** The expression of apoptotic proteins in wild-type SGC-7901 cells and SGC-7901 cells stably transfected with shASMase or shCtrl after rMV-Hu191 (MOI = 0.01) treatment for 72 h. *P < 0.05, **P < 0.01, ***P < 0.001

The representative microscopic images showed that cell density of rMV-Hu191 and combinational treatment was obviously increased in the presence of imipramine, an ASMase functional inhibitor (Fig. s5). And the ratio of apoptotic cells was prominently decreased after imipramine treatment (Fig. 3a). Imipramine treatment also markedly suppressed the cleavage of caspase-3 and PARP (Fig. 3b). To further clarify the participation of ASMase in rMV-Hu191- and combination-induced apoptosis, we constructed SGC-7901 cells stably knockdown ASMase protein levels using shRNA (Fig. 3c). As shown in Fig. 3d and Fig. s6, ASMase depletion caused remarkable reduction in rMV-Hu191-induced apoptosis and cytotoxicity in SGC-7901 cells. Taken together, these results confirmed the significance of ASMase in rMV-Hu191- and combination-induced apoptosis from the aspects of function and expression.

### rMV-Hu191 recruited ASMase to the lipid rafts

**Fig. 4:**
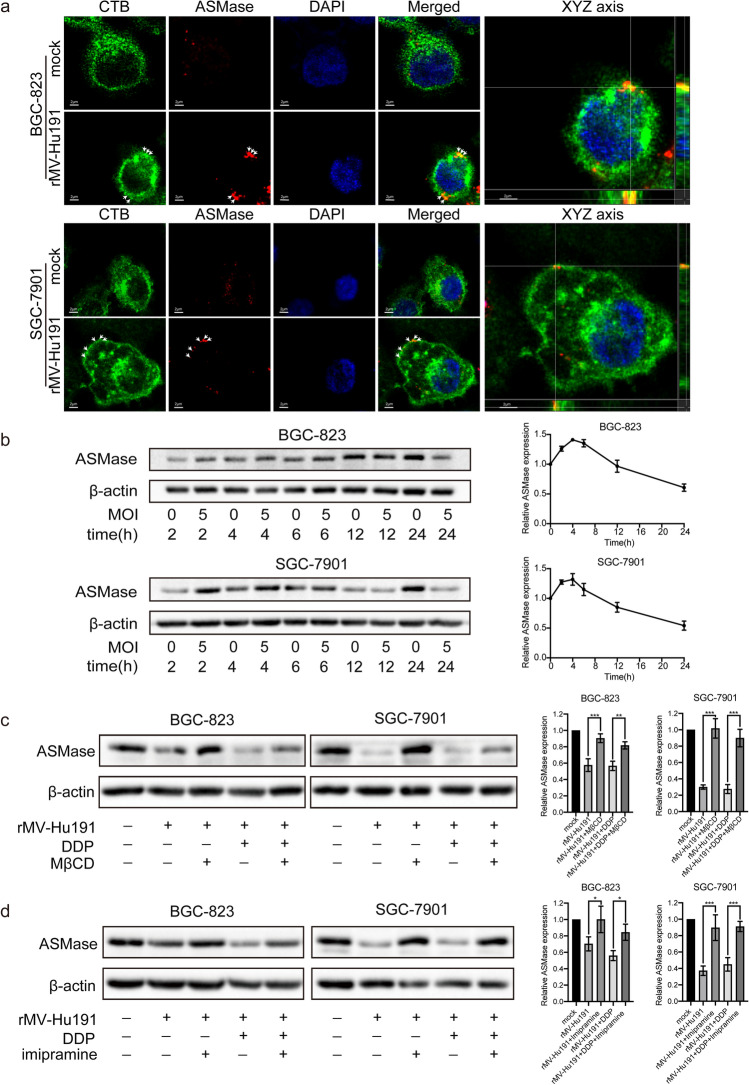
Transfer of ASMase to lipid rafts in rMV-Hu191-treated GC cells. **a** ASMase protein (red) was co-localized in CTB-labelled lipid rafts domains (green) after infection (MOI = 5) for 8 h exhibited by immunofluorescence in GC cells. White arrows indicated co-localization areas of the two probes. Further confirm of the co-localization by *Z*-axis scanning from the three-dimensional level. Scale bar = 2 μm. **b** The expression of ASMase in GC cells treated with rMV-Hu191 (MOI = 5) at the initial stage and the quantitative densitometry analysis from repeated blots. ASMase levels in BGC-823 and SGC-7901cells 72 h after the indicated treatments, with or without MβCD (**c**) and imipramine (**d**) coupled with the statistical significance based on triplicated WB data

According to the above results, we concluded that the antitumor effect was correlated with ASMase and lipid rafts, respectively. Therefore, we hypothesized that there existed certain relationship between ASMase and lipid rafts in GC cells with rMV-Hu191 treatment. As shown in Fig. 4a and Fig. s7, ASMase was found to be concentrated and co-localized with lipid rafts domains on plasma membranes after rMV-Hu191 infection for 8 h. The *Z*-axis scanning further confirmed the co-localization of ASMase and lipid rafts domains from the three-dimensional level. And the western blot results showed that after the rMV-Hu191 infection, the expression of ASMase increased at initial stage and then decreased (Fig. 4b and Fig. s8) with statistical differences. As shown in Fig. 4c, d and Fig. s9, the inhibition level of ASMase treated with rMV-Hu191 alone or combined treatment for 48 h was significantly reversed by culturing with MβCD and imipramine, respectively. In conclusion, the co-localization observed by immunofluorescence was consistent with the western blot results, and it revealed the translation of ASMase from cytoplasm to lipid rafts in GC cells infected with rMV-Hu191.

### Combined therapy synergized to suppress cell growth and increase apoptosis in a multi-drug-resistant signet ring cell GC cell line GCSR1

**Fig. 5:**
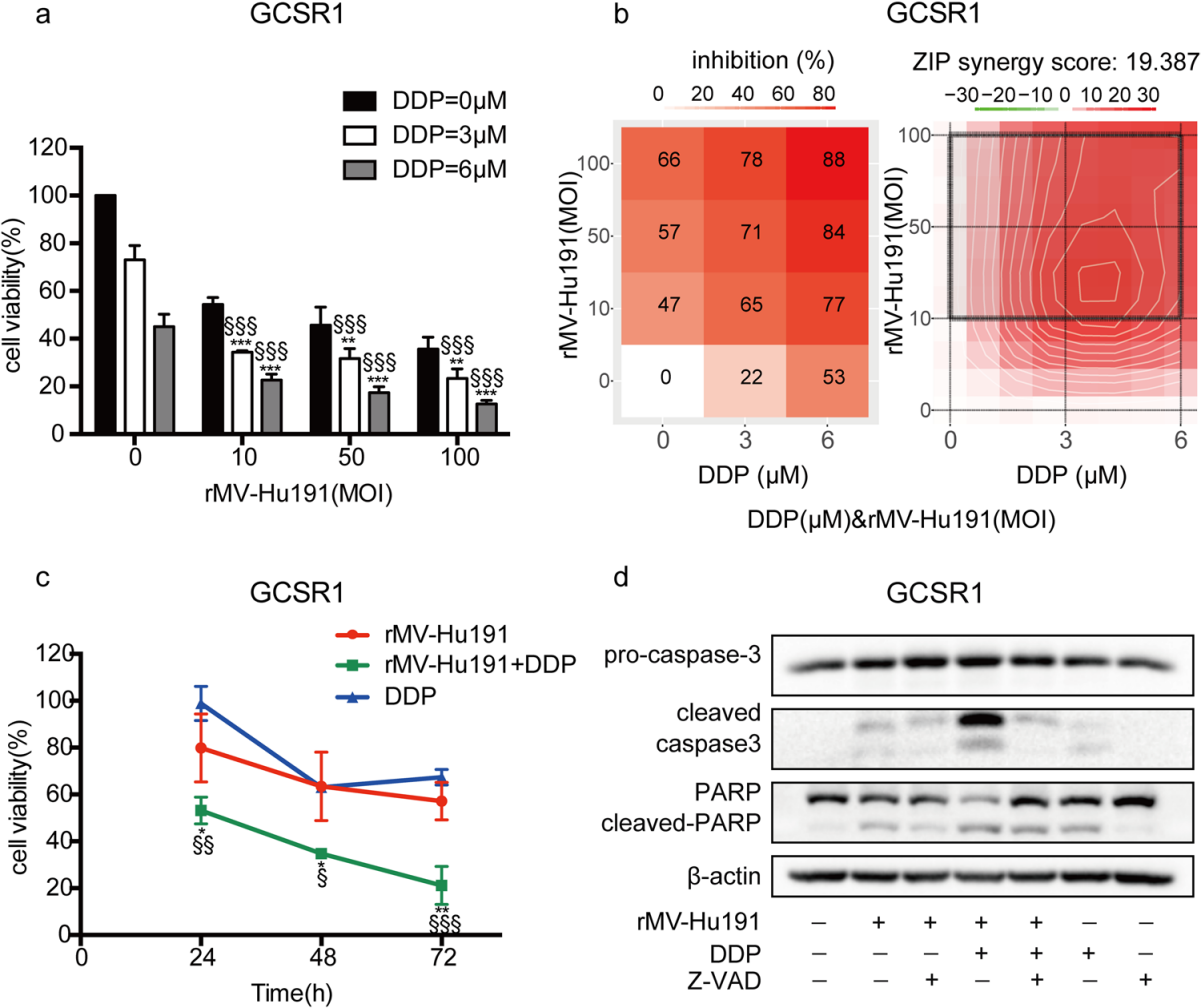
rMV-Hu191 and DDP inhibited cell growth and increased apoptosis synergistically in a multi-drug-resistant signet ring cell GC cell line GCSR1. **a** Viability of GCSR1 cells after combinational treatment with rMV-Hu191 and DDP for 72 h. Data presented as mean ± SE. **b** Inhibition and ZIP synergy score of rMV-Hu191 and DDP in GCSR1 cells. Circled area indicated the strongest synergistic region. **c** Growth curves of GCSR1 cells treated with rMV-Hu191 (MOI = 10) and DDP (6 μM). **d** Expression of apoptotic proteins in GCSR1 cells dealt with the synergistic treatment, in the presence or absence of Z-VAD (100 μM) for 72 h. * indicated comparison between the combinational- and rMV-Hu191-treated groups, *P < 0.05, **P < 0.01, ***P < 0.001 (one-way ANOVA); ^§^ indicated comparison between the combinational- and DDP-treated groups, ^§^*P* < 0.05, ^§§^*P* < 0.01, ^§§§^*P* < 0.001 (one-way ANOVA)

To evaluate the clinical potential of the combined therapy, a multi-drug resistant GC cell line GCSR1, established from a Chinese male signet ring cell gastric cancer patient, which is resistant to cisplatin, 5-fluorouracil and mitomycin, was treated with rMV-Hu191 and DDP. Coincided with results of BGC-823 and SGC-7901 cells, combined therapy generated synergistic cytotoxicity in GCSR1 cells as well (Fig. 5a). The strongest synergistic effect was found within the region of dose combinations (MOI = 10 and DDP = 3 μM) with average ZIP synergy scores 19.387 (Fig. 5b). After treatment with optimum doses, the synergy was initially observed at 24 h with time-dependent manner (Fig. 5c). Furthermore, the combined treatment also dramatically augmented the caspase-dependent apoptotic cell death in GCSR1 (Fig. 5d).

### Combinational treatment increased antitumor activity in human GC xenografts with trivial side-effects

**Fig. 6:**
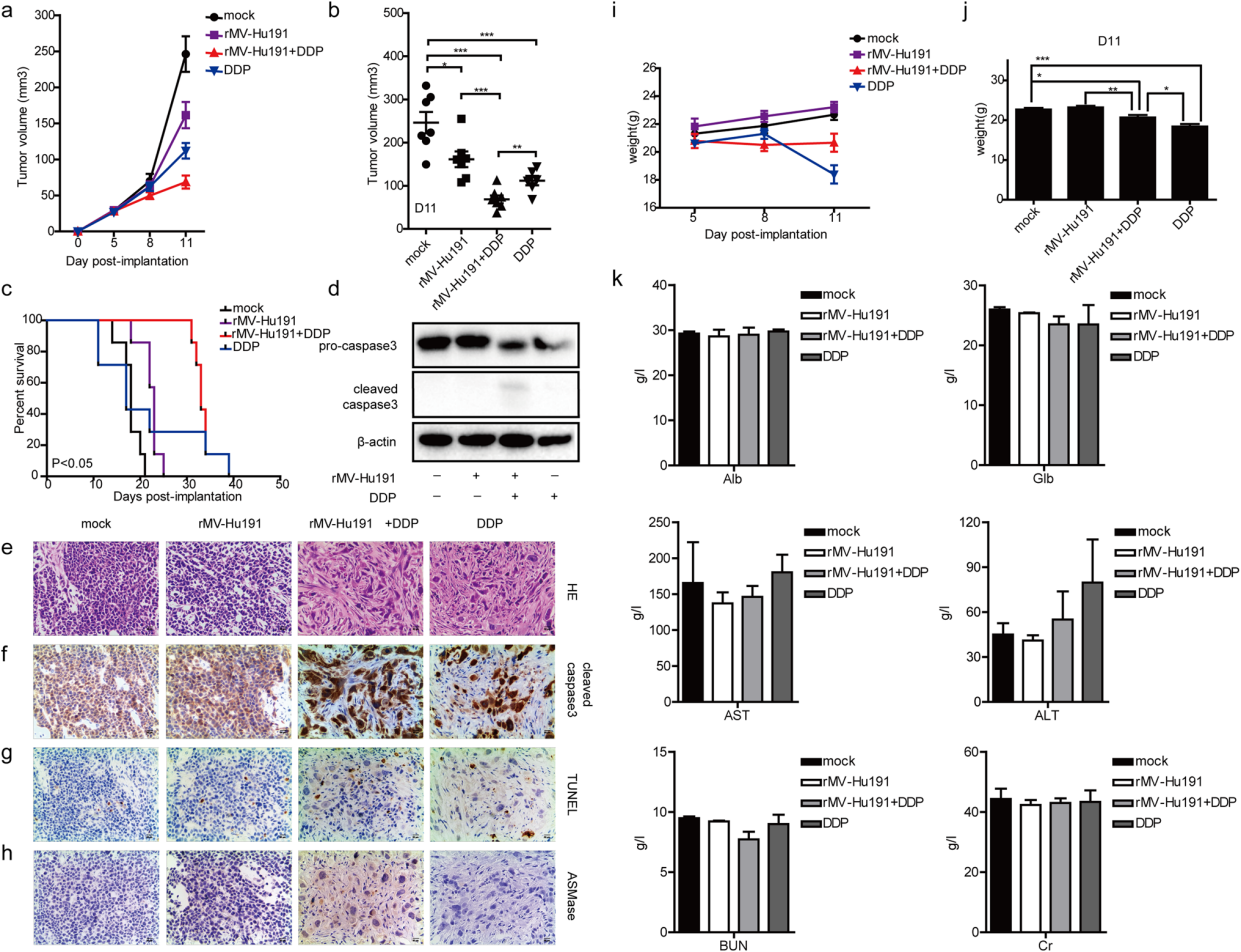
Combinational treatment of rMV-Hu191 with DDP increased antitumor activity in vivo with trivial side-effects. **a** Tumor growth in mice bearing GC xenografts with desired treatments. **b** Difference in tumor volume on post-implantation day 11. *P < 0.05, **P < 0.01, ***P < 0.001 (unpaired *t*-test). **c** The survival rate of tumor-bearing mice. *P* < 0.05 (log-rank test). **d** Expression of pro-caspase-3 and cleaved-caspase-3 in protein extracts from tumor tissues. HE staining (**e**), IHC of cleaved-caspase-3 (**f**) and ASMase (**h**), TUNEL (**g**) assays in tumor tissues. Scale bar = 50 μm. **i**, **j** Body weight of mice bearing BGC-823 xenografts. **k** Evaluation of liver and kidney functions in mice. *P < 0.05, **P < 0.01, ***P < 0.001 (one-way ANOVA)

Human GC xenografts were successfully established in nude mice to assess the in vivo effects of the combinational treatment. After 11-day treatment, the tumor regression effects were observed stronger in combined group than rMV-Hu191 group or DDP group (Fig. 6a, b). Administration of combinational treatment (median survival = 33 days) resulted significantly prolonged survival compared with rMV-Hu191 (median survival = 23 days) or DDP (median survival = 17 days) treatment alone (Fig. 6c). H&E staining from combined group showed distinct signs of tumor mass degradation, with large necrotic and apoptotic areas containing tumor infiltrating cytoplasmic eosinophilia (Fig. 6e). Expression of apoptotic cells was considerably higher in co-treated tumors by detecting cleaved caspase 3 and in situ apoptosis using western blotting (Fig. 6d), IHC (Fig. 6f) and TUNEL analysis (Fig. 6g). And the expression level of ASMase was significantly increased by the rMV-Hu191 and combined treatment (Fig. 6h). Remarkably, the administration of DDP resulted in an obvious weight loss on day 11, whereas the combinational treatment was well tolerant, suggesting that rMV-Hu191 could attenuate the side-effects of DDP (Fig. 6i, j). We further validated levels of Alb, Glb, AST, ALT, BUN, and Cr in angular vein blood to perform liver and kidney functions, and demonstrated that there was no statistical difference between treatment and control groups (Fig. 6k). Taken together, these results indicated that rMV-Hu191 could synergize the effects of DDP to inhibit tumor growth in vivo by augmenting apoptosis accompanying by trivial side-effects, which was in accordance with the mechanism in vitro.

## Discussion

To increase the sensitization of chemotherapy, combination therapies of oncolytic MV Edmonston strain and chemotherapy and radiotherapy have been extensively explored [38–40]. Oncolytic MV in combination with chemotherapeutics is more promising, since both primary tumors and metastases can be efficiently treated by such regimens. With the same advantage as a MV strain, the safety of Chinese Hu-191 strain derivatives has been proved in the application of vaccination in China [41]. Our study is the first to prove that combination therapy of oncolytic MV Chinese Hu-191strain and DDP could serve as a promising strategy in GC treatment. From our data, rMV-Hu191 in combination with DDP synergistically induced stronger antitumor effect than rMV-Hu191 or DDP alone in two human GC cell lines and one multi-drug-resistant GC cell line. Importantly, ZIP analysis revealed synergistic activity of this chemovirotherapy strategy, with higher average synergy scores in GSCR1 cells than that of SGC-7901 and BGC-823 cells. In literature reports, whether chemical drug would hamper virus replication lacked consistency [42, 43]. However, in our study, there was almost no alternation of virus growth kinetics in GC cells treated with rMV-Hu191 alone or combination with DDP. In vivo studies confirmed that intratumoral rMV-Hu191 injection combined with intraperitoneal DDP injection resulted in a significant reduction in tumor growth compared with rMV-Hu191 alone or DDP alone injection, and prolonged the survival of human GC xenografts significantly. And the body weight of the combination group was slightly heavier than that of the DDP group, indicating that the toxicity of DDP could be partially reduced by combination of rMV-Hu191.

Apoptosis, known as programed cell death, is controlled by genes and a series of enzymes [44], and has been recognized as the common fate of cancer cells during therapy [45]. DDP can induce apoptosis in GC cells [46]. In this study, we found that rMV-Hu191 combined with DDP exhibited significant antitumor effect against GC through induction of caspase-dependent apoptosis both in vitro and vivo. In both two GC cell lines and one multi-drug-resistant GC cell line, the ratio of apoptotic cells and the expression of activated caspase 3 and PARP were significantly increased in combined group compared to rMV-Hu191 or DDP alone group. Furthermore, the pan-caspase inhibitor Z-VAD could partially reverse the induction of apoptotic cell death after rMV-Hu191 or combinational treatment. In BGC-823 xenografts, induction of caspase-dependent apoptosis was approved by in situ detection of caspase 3 activation and TUNEL assay. Therefore, the capability to induce caspase-dependent apoptosis was the major mechanism for rMV-Hu191 combined DDP to synergistically inhibit GC.

Lipid rafts were specific regions of membranes which were rich in sphingolipids and cholesterols [47], and were involved in many cellular processes including signal transduction, membrane trafficking, skeletal reconstruction [48]. Lipid rafts disruption following treatment with the MβCD, a most common cholesterol-depleting agent, inhibited both rMV-Hu191-induced and rMV-Hu191 plus DDP-induced apoptosis in the two GC cell lines (Fig. 2). These results indicated that lipid rafts could influence rMV-Hu191- and combination-induced apoptosis.

It is well known that ASMase can hydrolyze SM to generate ceramide [49]. Ceramide, a bioactive sphingolipid, was a key regulator in the initiation step of apoptosis [50].

When plasma membrane was wounded by exposure to the bacterial or virus, the lysosomal ASMase can be delivered to the outer leaflet of the plasma membrane, the later event would stimulate ceramide generation and apoptosis initiation [51]. From our data, the expression of ASMase increased at initial stage in vitro and in vivo and then decreased in vitro (Figs. 4b, 6h). Inhibition of ASMase activation by imipramine, an inhibitor of acid sphingomyelinase, could reduce the ratio of apoptotic cells and the expression of activated caspase 3 and PARP in rMV-Hu191 and rMV-Hu191 plus DDP treated GC (Fig. 3). Moreover, reduction the level of ASM by shRNA, could also reverse the rMV-Hu191-induced and rMV-Hu191 plus DDP-induced of apoptotic cell death (Fig. 3). To verify an interaction between ASMase and lipid rafts, immunofluorescence analyses were performed. According to the obtained results, co-localization of ASMase with the lipid rafts marker CTB was visible after early infection of rMV-Hu191 (Fig. 4a) and the expression level of ASMase within GC cell could be reversed by MβCD and imipramine (Fig. 4c, d). Our data suggested that ASMase transferred from cytosol to plasma membrane lipid raft microdomains in the early stage and played a central role in rMV-Hu191- and combination-induced GC apoptosis.

**Fig. 7:**
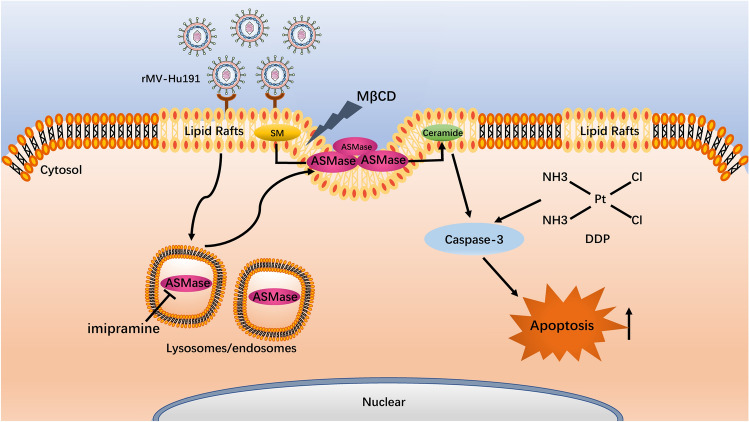
Schematic illustration of the combination of rMV-Hu191- and DDP-induced antitumor effects

Based on the above data, our findings are consistent with the following mechanism (Fig. 7): First of all, the ASMase within cytoplasmic was activated and translocated to plasma membrane lipid raft microdomains during early stages of rMV-Hu191 infection. The later phase is based on the integrity of lipid raft microdomains and the ceramide induction might be involved it. Lastly, rMV-Hu191 and DDP cooperated to trigger caspase-dependent apoptosis in GC cells.

In conclusion, our findings suggested that combination of rMV-Hu191 with DDP chemotherapy has potent and synergistic therapeutic efficacy against human GC both in vivo and in vitro by ASMase-mediated apoptosis requiring integrity of lipid raft microdomains. Due to the relatively low side effects of rMV-Hu191, co-administration of rMV-Hu191 could serve as a safe and effective strategy. Taken together, the combinational chemovirotherapy investigated here could potentially serve as an adjuvant therapy against human GC.

## Supplementary Information

Below is the link to the electronic supplementary material.

Fig. s1 The anti-proliferative effect of rMV-Hu191 combined with DDP in GC cell lines. Representative morphological changes of BGC-823 and SGC-7901 cells after combinational treatment for different periods of time. Scale bar = 200 μm (TIF 9180 KB)

Fig. s2 DDP did not enhance rMV-Hu191 replication in GC cells. MV-P protein expression treated with rMV-Hu191 or combinational therapy was determined by immunofluorescence assay. Scale bar = 100 μm (TIF 9642 KB)

Fig. s3 Treatment of MβCD solubilized lipid rafts. Distribution of flotillin 1 a marker protein of lipid rafts throughout the density gradient (fractions labelled 1 to 10 from top to bottom) and the insoluble pellet (P) with or without MβCD treatment in BGC-823 and SGC-7901 cells. Circled area indicates the location of lipid rafts in the gradient (TIF 5199 KB)

Fig. s4 The antitumor capability of rMV-Hu191 combined with DDP was reversed by MβCD treatment. Representative images showing that MβCD treatment reversed the cell density of rMV-Hu191 combined with DDP in GC cells (TIF 9635 KB)

Fig. s5 Imipramine treatment inhibited the cytotoxicity of rMV-Hu191 and combinational treatment in GC cells. Representative images of GC cells cultured with rMV-Hu191 and DDP for 48 h, with or without imipramine treatment (TIF 9286 KB)

Fig. s6 Knockdown of ASMase inhibited cytotoxicity of rMV-Hu191. Morphological changes of SGC-7901 cells stably transfected with shASMase or shCtrl 72 h after rMV-Hu191 infection (MOI = 0.01) (TIF 9330 KB)

Fig. s7 Co-localization of ASMase and lipid rafts after rMV-Hu191 infection. BGC-823 cells and SGC-7901 cells were infected with rMV-Hu191 (MOI = 5) for 8 h. ASMase (red) and lipid rafts (green) were observed co-localized in the plasma membranes (white arrows) (TIF 8209 KB)

Fig. s8 Additional Western blotting gels for ASMase and the statistical analysis of Fig 4b. Additional Western blottings for ASMase at different time points of rMV-Hu191 infection and the quantitative densitometry analysis from repeated blots (TIF 7597 KB)

Fig. s9 Repeated Western blotting gels for ASMase and the statistical analysis of Fig 4c,d. (a,b) Repeated Western blottings for ASMase after the indicated treatments with or without MβCD and imipramine, and the statistical significance based on triplicated WB data. *P<0.05, **P<0.01, ***P<0.001 (TIF 8015 KB)

## Abbreviations

GC: Gastric cancer
DDP: Cisplatin
rMV-Hu191: Recombinant Hu191 strain of measles virus
MV: Measles virus
ASMase: Acid sphingomyelinase
SM: Sphingomyelin
MβCD: Methyl-β-cyclodextrin
CTB: Cholera toxin subunit B
CCK-8: Cell counting kit-8
ZIP: Zero interaction potency
FBS: Fetal bovine serum
ATCC: American Type Culture Collection
DMEM: Dulbecco’s modified Eagle’s medium
Z-VAD: Z-VAD-FMK
MOI: Multiplicity of infection
IC50: Half maximal inhibitory concentration
TCID 50: The median tissue culture infective dose
DMSO: Dimethyl sulfoxide
PFA: Paraformaldehyde
MV-P: Anti-measles phosphoprotein
Alb: Albumin
Glb: Globulin
AST: Aspartate aminotransferase
ALT: Alanine aminotransferase
BUN: Blood urea nitrogen
Cr: Creatine
IF: Immunofluorescence
IHC: Immunohistochemistry
H&E: Hematoxylin and eosin
