# Supplementary figures and images for: Synergism of rMV-Hu191 with cisplatin to treat gastric cancer by acid sphingomyelinase-mediated apoptosis requiring integrity of lipid raft microdomains
Source: Gastric Cancer. 2021 Jul 12;24(6):1293–306. doi: 10.1007/s10120-021-01210-8 (PMC8502160; doi:10.1007/s10120-021-01210-8)

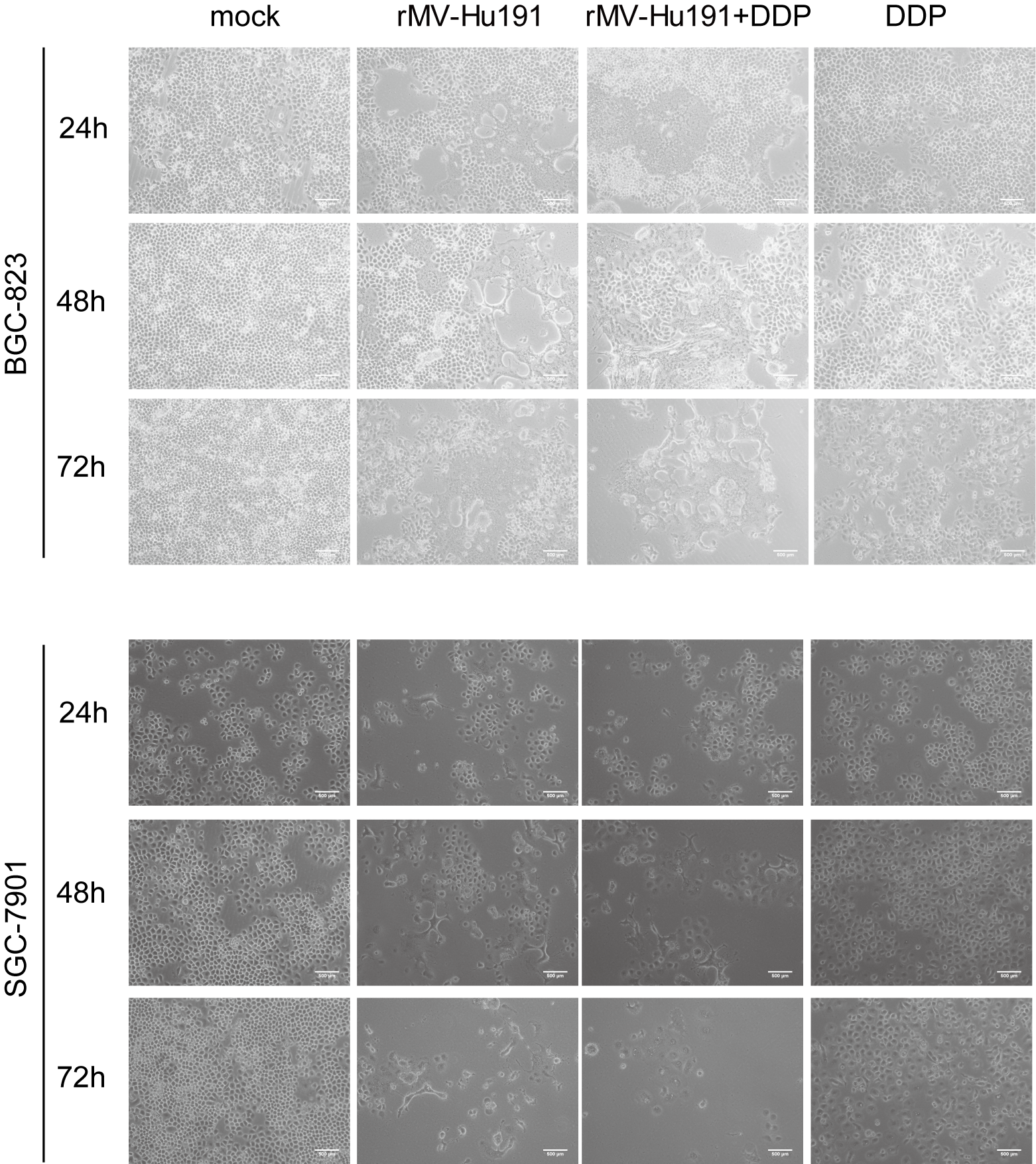

Supplement: Supplementary file 1 — The anti-proliferative effect of rMV-Hu191 combined with DDP in GC cell lines. Representative morphological changes of BGC-823 and SGC-7901 cells after combinational treatment for different periods of time. Scale bar = 200 μm (TIF 9180 KB) [file 10120_2021_1210_MOESM1_ESM.tif]

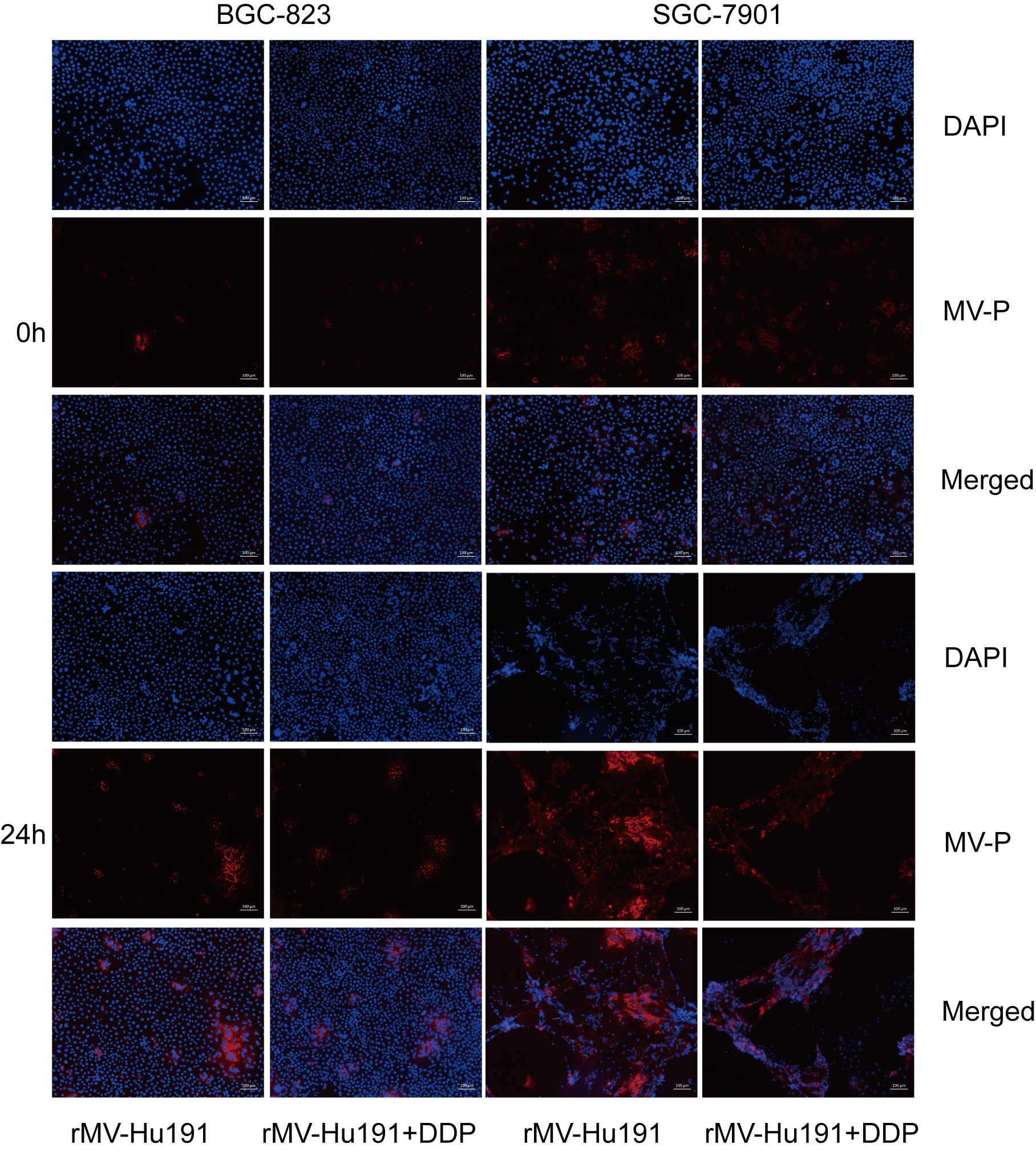

Supplement: Supplementary file 2 — DDP did not enhance rMV-Hu191 replication in GC cells. MV-P protein expression treated with rMV-Hu191 or combinational therapy was determined by immunofluorescence assay. Scale bar = 100 μm (TIF 9642 KB) [file 10120_2021_1210_MOESM2_ESM.tif]

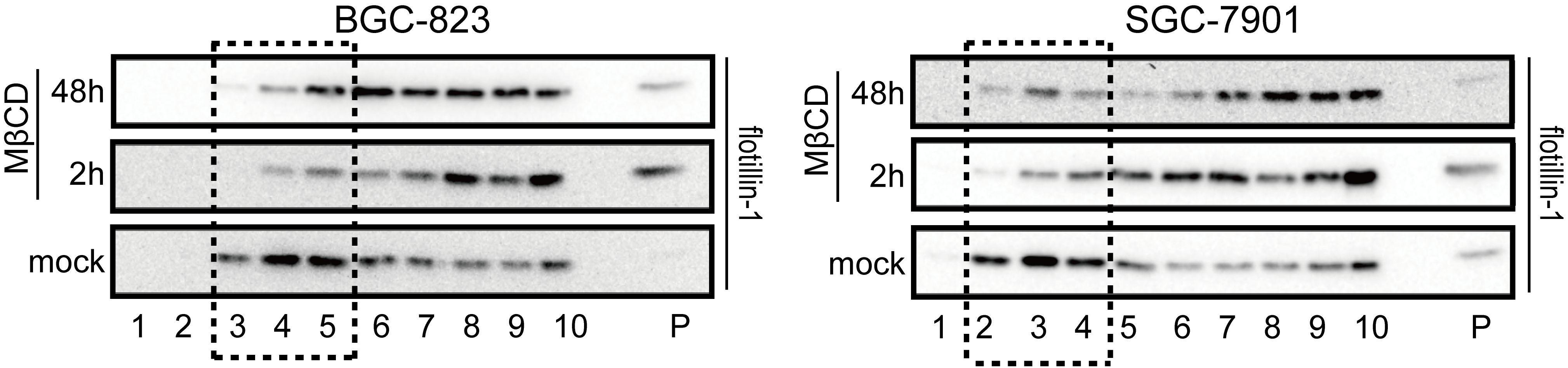

Supplement: Supplementary file 3 — Treatment of MβCD solubilized lipid rafts. Distribution of flotillin 1 a marker protein of lipid rafts throughout the density gradient (fractions labelled 1 to 10 from top to bottom) and the insoluble pellet (P) with or without MβCD treatment in BGC-823 and SGC-7901 cells. Circled area indicates the location of lipid rafts in the gradient (TIF 5199 KB) [file 10120_2021_1210_MOESM3_ESM.tif]

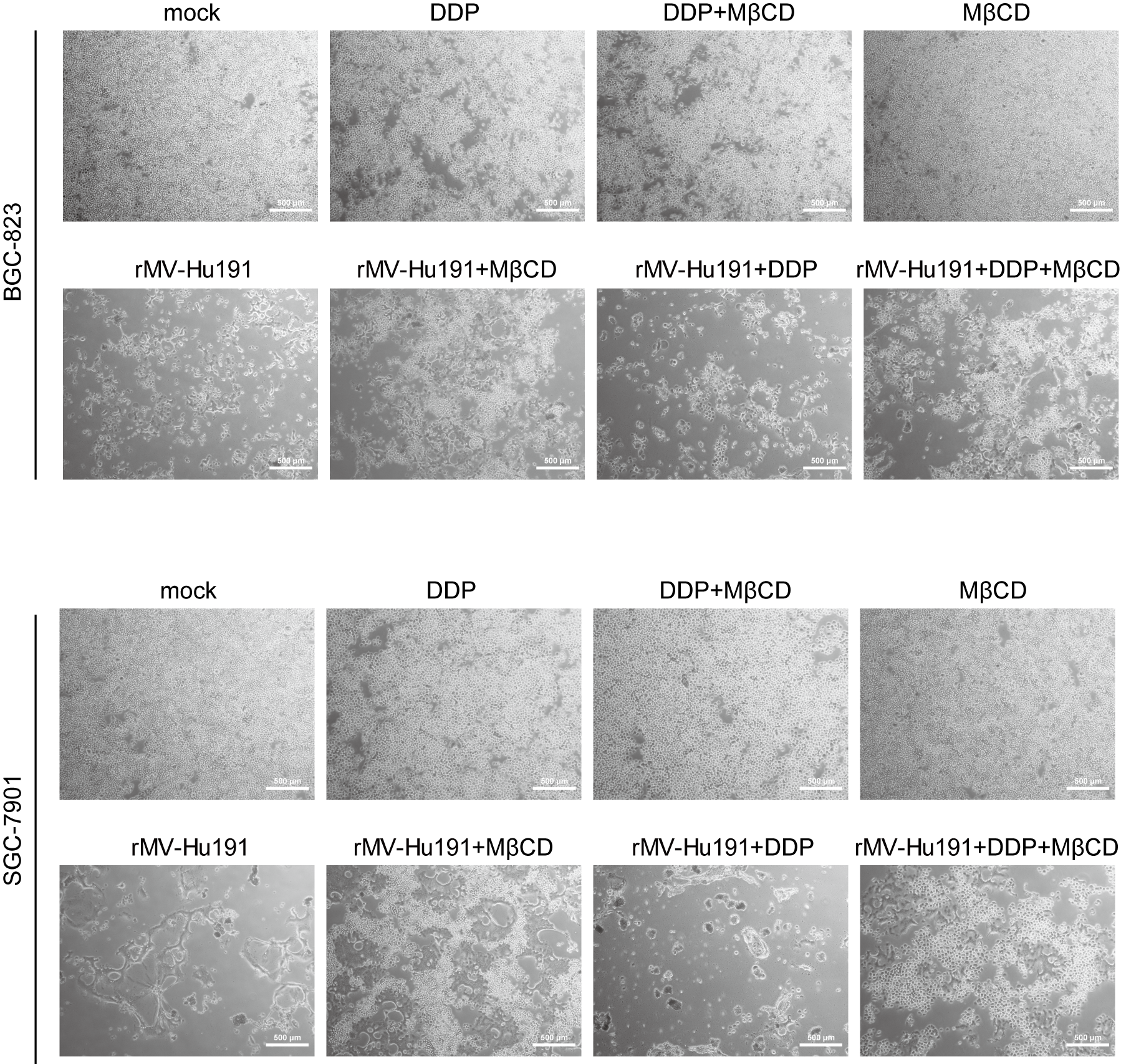

Supplement: Supplementary file 4 — The antitumor capability of rMV-Hu191 combined with DDP was reversed by MβCD treatment. Representative images showing that MβCD treatment reversed the cell density of rMV-Hu191 combined with DDP in GC cells (TIF 9635 KB) [file 10120_2021_1210_MOESM4_ESM.tif]

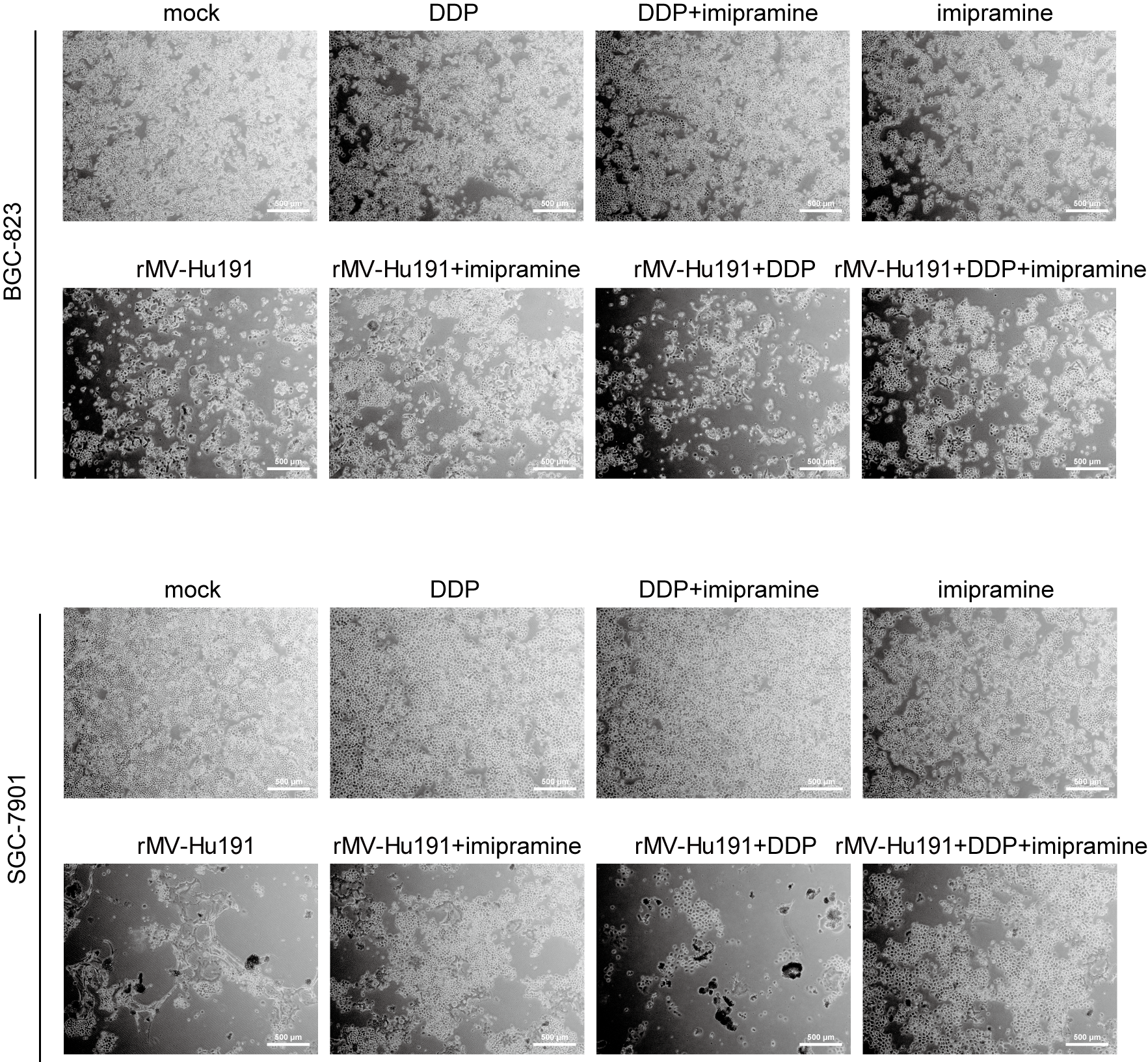

Supplement: Supplementary file 5 — Imipramine treatment inhibited the cytotoxicity of rMV-Hu191 and combinational treatment in GC cells. Representative images of GC cells cultured with rMV-Hu191 and DDP for 48 h, with or without imipramine treatment (TIF 9286 KB) [file 10120_2021_1210_MOESM5_ESM.tif]

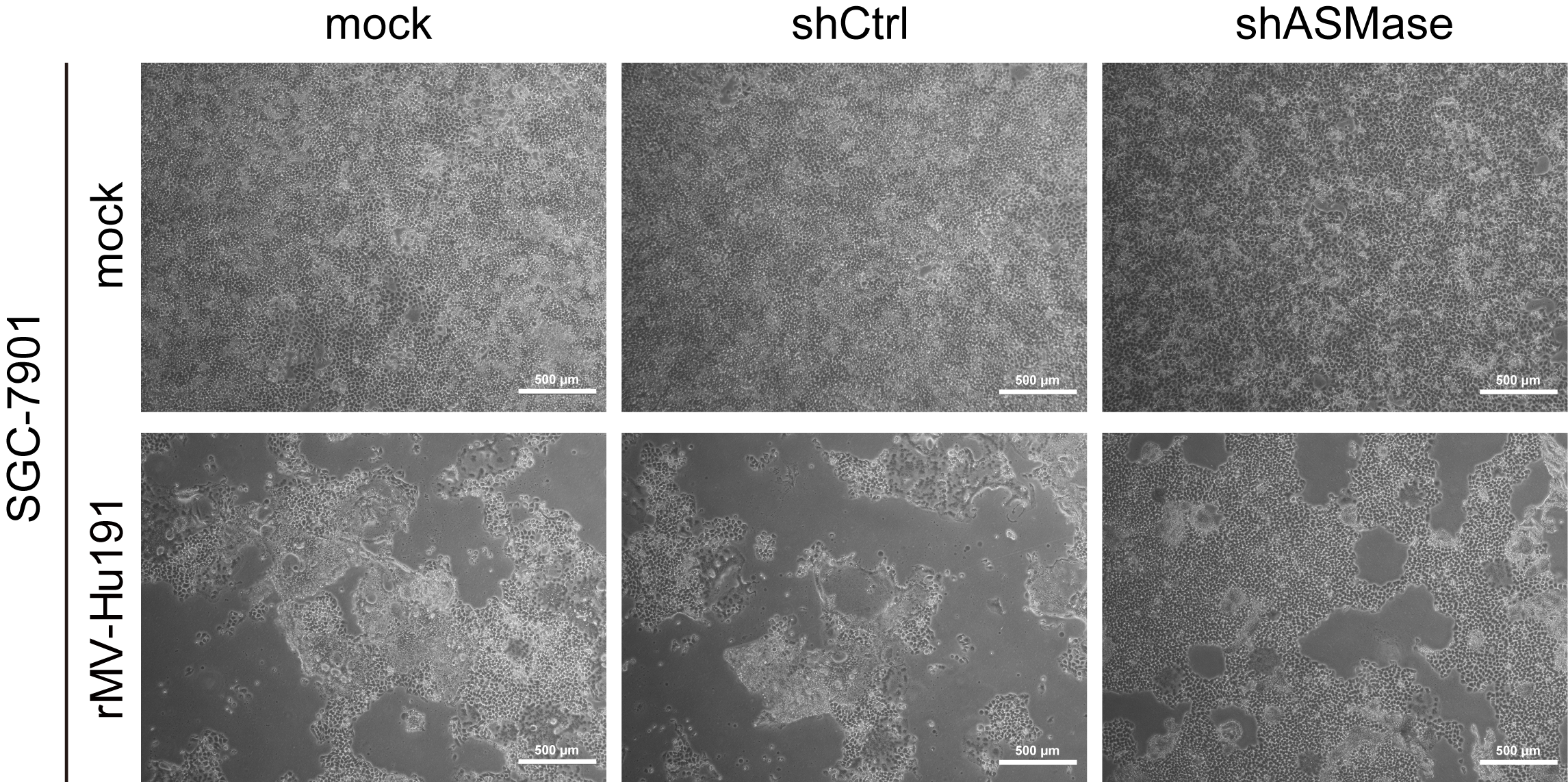

Supplement: Supplementary file 6 — Knockdown of ASMase inhibited cytotoxicity of rMV-Hu191. Morphological changes of SGC-7901 cells stably transfected with shASMase or shCtrl 72 h after rMV-Hu191 infection (MOI = 0.01) (TIF 9330 KB) [file 10120_2021_1210_MOESM6_ESM.tif]

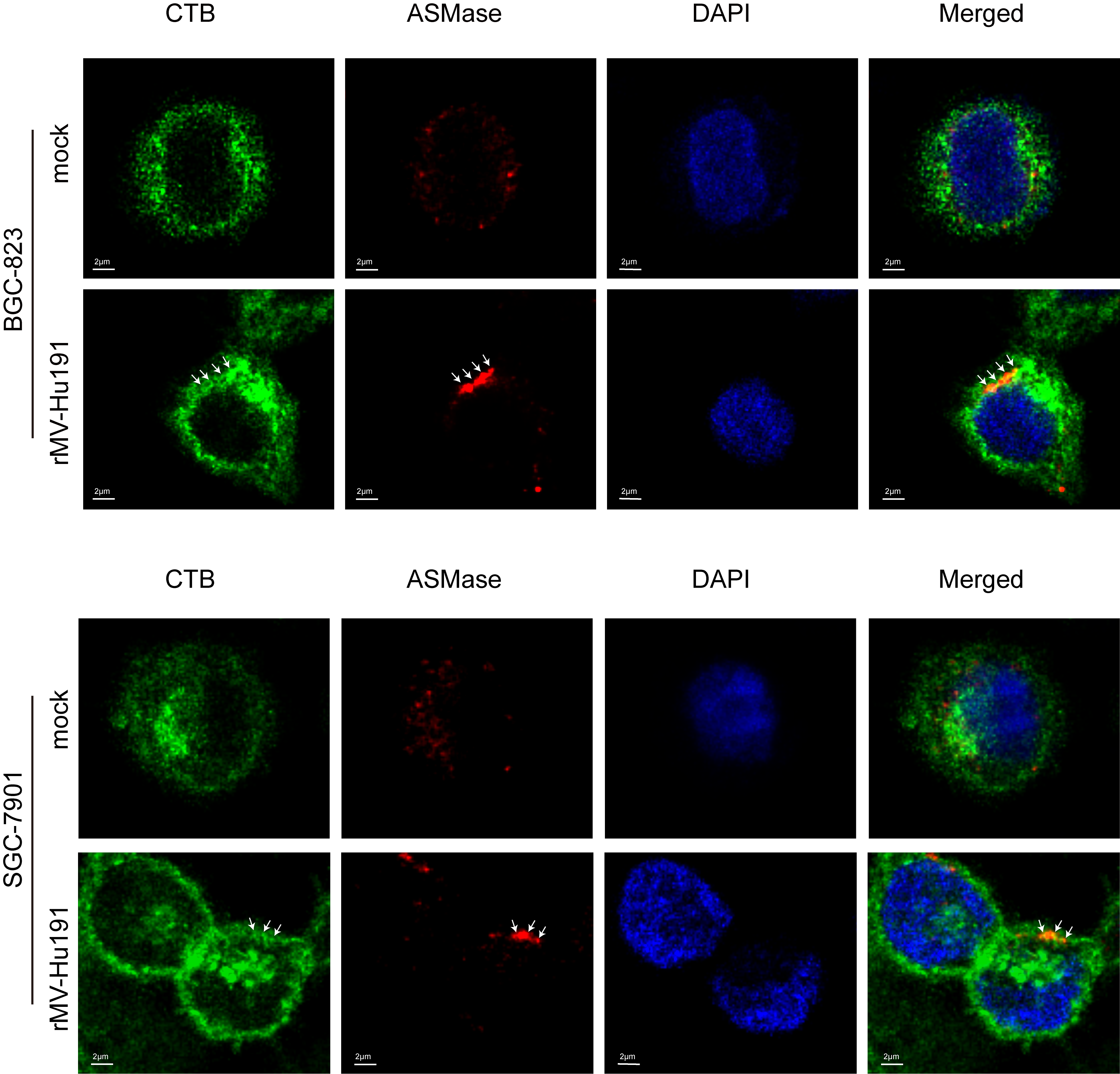

Supplement: Supplementary file 7 — Co-localization of ASMase and lipid rafts after rMV-Hu191 infection. BGC-823 cells and SGC-7901 cells were infected with rMV-Hu191 (MOI = 5) for 8 h. ASMase (red) and lipid rafts (green) were observed co-localized in the plasma membranes (white arrows) (TIF 8209 KB) [file 10120_2021_1210_MOESM7_ESM.tif]

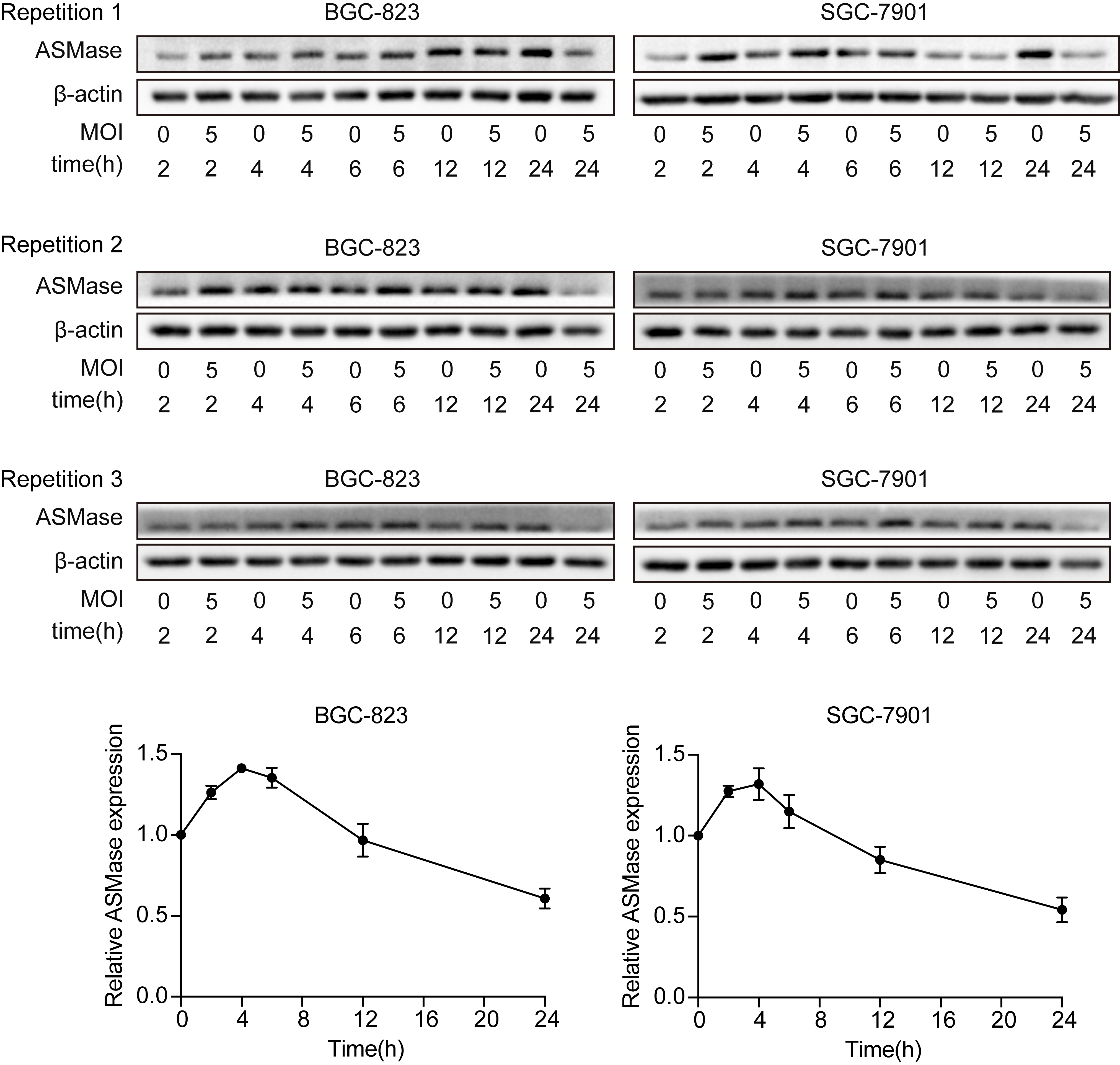

Supplement: Supplementary file 8 — Additional Western blotting gels for ASMase and the statistical analysis of Fig 4b. Additional Western blottings for ASMase at different time points of rMV-Hu191 infection and the quantitative densitometry analysis from repeated blots (TIF 7597 KB) [file 10120_2021_1210_MOESM8_ESM.tif]

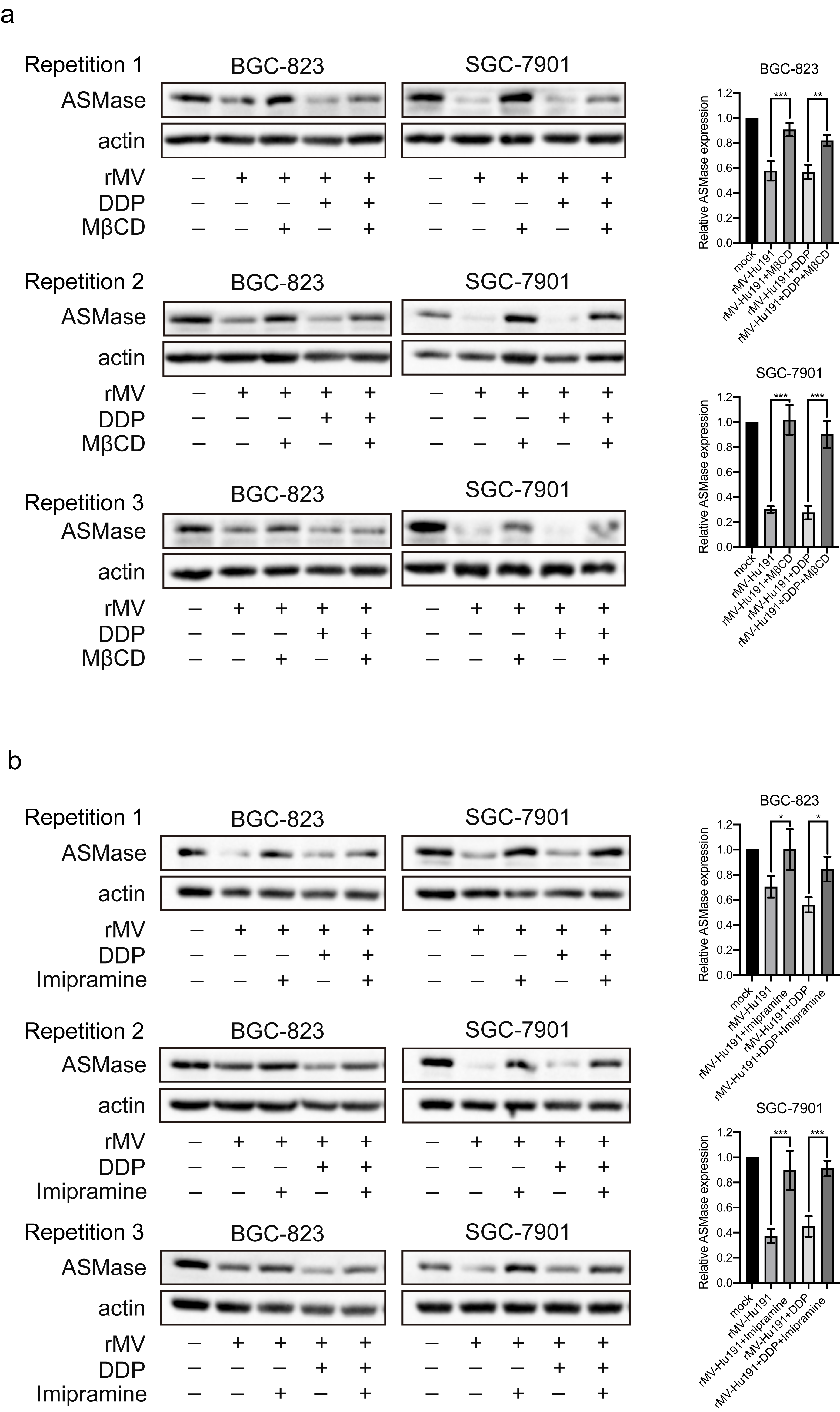

Supplement: Supplementary file 9 — Repeated Western blotting gels for ASMase and the statistical analysis of Fig 4c,d. (a,b) Repeated Western blottings for ASMase after the indicated treatments with or without MβCD and imipramine, and the statistical significance based on triplicated WB data. *P<0.05, **P<0.01, ***P<0.001 (TIF 8015 KB) [file 10120_2021_1210_MOESM9_ESM.tif]
